# Identification and Management of Statin-Associated Symptoms in Clinical Practice: Extension of a Clinician Survey to 12 Further Countries

**DOI:** 10.1007/s10557-017-6727-0

**Published:** 2017-05-03

**Authors:** Robert S. Rosenson, Shravanthi R. Gandra, Jan McKendrick, Ricardo Dent, Heather Wieffer, Lung-I Cheng, Alberico L. Catapano, Paul Oh, G. Kees Hovingh, Erik S. Stroes

**Affiliations:** 1 Icahn School of Medicine at Mount Sinai, 1425 Madison Ave, MC1 Level, New York, NY 10029 USA; 2 Amgen Inc., Thousand Oaks, CA USA; 3 PRMA Consulting, Fleet, Hampshire UK; 4 University of Milano and IRCCS Multimedica, Milan, Italy; 5 Toronto Rehabilitation Institute, Toronto, ON Canada; 6 Academic Medical Center, Amsterdam, the Netherlands

**Keywords:** Hypercholesterolemia, Statin-associated muscle symptoms, Statin-associated symptoms, Reduced statin tolerance, Clinical practice

## Abstract

**Purpose:**

Statins are the first-choice pharmacological treatment for patients with hypercholesterolemia and at risk for cardiovascular disease; however, a minority of patients experience statin-associated symptoms (SAS) and are considered to have reduced statin tolerance. The objective of this study was to establish how patients with SAS are identified and managed in clinical practice in Austria, Belgium, Colombia, Croatia, the Czech Republic, Denmark, Portugal, Switzerland, Russia, Saudi Arabia, Turkey, and the United Arab Emirates.

**Methods:**

A cross-sectional survey was conducted (2015–2016) among clinicians (*n* = 60 per country; Croatia: *n* = 30) who are specialized/experienced in the treatment of hypercholesterolemia. Participants were asked about their experience of patients presenting with potential SAS and how such patients were identified and treated.

**Results:**

Muscle-related symptoms were the most common presentation of potential SAS (average: 51%; range across countries [RAC] 17–74%); other signs/symptoms included persistent elevation in transaminases. To establish whether symptoms are due to statins, clinicians required rechallenge after discontinuation of statin treatment (average: 77%; RAC 40–90%); other requirements included trying at least one alternative statin. Clinicians reported that half of high-risk patients with confirmed SAS receive a lower-dose statin (average: 53%; RAC 43–72%), and that most receive another non-statin lipid-lowering therapy with or without a concomitant statin (average: 65%; RAC 52–83%).

**Conclusions:**

The specialists and GPs surveyed use stringent criteria to establish causality between statin use and signs or symptoms, and persevere with statin treatment where possible.

**Electronic supplementary material:**

The online version of this article (doi:10.1007/s10557-017-6727-0) contains supplementary material, which is available to authorized users.

## Background

Statins are the first-choice pharmacological treatment to reduce circulating levels of low-density lipoprotein cholesterol (LDL-C), an important and modifiable risk factor for cardiovascular disease [1, 2]. The incidence of reported adverse events attributed to statins is low in clinical trials [3, 4]; however, in observational studies, approximately 10% of patients experience side effects (statin-associated symptoms [SAS]) [5–8]. The most commonly reported SAS are statin-associated muscle symptoms (SAMS), such as muscle discomfort or weakness [9–11]; other less common presentations that may be attributed to SAS include hepatic, gastrointestinal, or central nervous system (CNS) effects [12].

Patients with SAS may require a decrease in dose or complete discontinuation of statin therapy [12]. This limits effective treatment, putting patients at increased risk of cardiovascular morbidity and mortality [13, 14]. National and international clinical guidelines have been developed in recent years to help clinicians evaluate and manage patients with SAS in clinical practice [2, 9–12, 15, 16]. Such guidelines provide pragmatic definitions of SAMS, and SAS in general, which include new approaches using the terminology “goal-inhibiting statin intolerance” that emphasize the effect of such symptoms on the ability to achieve lipid-lowering goals through adequate therapy or optimally reduce cardiovascular risk [9].

Even with the publication of guidelines, it remains unclear how clinicians manage patients presenting with SAS in clinical practice. In 2014, we conducted a survey of clinicians in 13 countries to establish how patients with SAS are identified and managed in clinical practice [17]. In 2015–2016, this work was extended to include a further 12 countries, to gain a broader understanding of treatment practice across additional countries and regions.

## Material and Methods

This cross-sectional survey was conducted among clinicians who specialize in the treatment of patients with hypercholesterolemia in Austria, Belgium, Colombia, Croatia, the Czech Republic, Denmark, Portugal, Switzerland, Russia, Saudi Arabia, Turkey, and the United Arab Emirates (UAE). The survey was conducted between December 2015 and August 2016.

### Questionnaire Development and Administration

The questionnaire was based on the one used in 2014 for the initial 13 countries [17]. The original questionnaire was reviewed by a clinical advisor before face validity was assessed through interviews with clinicians in seven of the original scope countries to ensure the questions were relevant, comprehensible, unambiguous, and asked only a single question. Analysis of the 2014 survey data served as further validation of the questionnaire. Minor modifications were made to the original questionnaire used in 2014 following discussions with clinicians, to account for the publication of clinical guidelines on SAS since the original survey and to improve the clarity of some questions. The participating clinicians were asked about their clinical experience of patients presenting with potential SAS, how patients with SAS were confirmed, and what treatment was subsequently started in these patients. No further validation was undertaken. Clinicians were asked to answer the questionnaire based on recollection of their experience in routine clinical practice. A summary of the questionnaire is available in the Supplementary Materials.

The survey was provided to clinicians in their local language using a web-based platform in all countries except Saudi Arabia and the UAE, where clinicians completed the survey at a face-to-face appointment with a researcher (clinician responses to survey questions were entered by the researcher into an offline copy of the survey). A pilot phase was conducted in all countries except Croatia; for this phase, two clinicians in each country completed the survey and provided feedback in a telephone interview. This feedback was used to confirm the face validity of the questionnaire in the local language, and to identify country-specific items to incorporate into the questionnaire (e.g., national guidelines).

Recruitment and data collection were performed by agencies specializing in healthcare fieldwork; clinicians were recruited by invitations sent to a panel of clinicians who had previously agreed to participate in healthcare research surveys. All participants were remunerated for their time at the fair market value rate prevalent in their country.

### Participants

Eligible clinicians had to be specialists (mainly cardiologists, with a small number of endocrinologists also recruited in Russia, Saudi Arabia, and the UAE to reflect local practices) or general/family physicians (GPs) who had practiced for at least 2 years since completing their specialist training. Specialists had to have treated at least 75 patients with hypercholesterolemia with pharmacological therapies in the previous 12 months. GPs had to have treated at least 50 such patients; this requirement was reduced to 25 in Croatia, the Czech Republic, and Denmark following feedback that this criterion was higher than the typical caseload of GPs in these countries. Clinicians were eligible to participate if they had treated at least five patients with statin intolerance in the previous 12 months.

The numbers of GPs and specialists varied across countries. The minimum number of specialists was pre-determined depending on the available pool of clinicians, and more GPs were recruited in countries where the minimum number of specialists was lower. Clinicians were recruited from different regions in each country.

### Statistical Analysis

All data were analyzed separately for each country according to a pre-specified statistical analysis plan. Analyses used descriptive statistics only since the survey was not designed or powered to make statistical comparisons between countries. In addition, exploratory analyses were planned to report results separately by clinician type (specialists and GPs).

All analyses were carried out using the statistical software package SAS (version 9.2 or later) and were independently verified by a statistician.

## Results

### Demographics of Study Participants

In total, 2002 clinicians were screened for participation in the survey; of these, 1003 (50%) met the eligibility criteria and 690 (69% of eligible clinicians) completed the survey. The planned sample size of 60 per country was met in all countries except Croatia, where 30 clinicians (15 specialists and 15 GPs) were recruited.

The number of specialists and GPs who completed the survey in each country is shown in Table 1. All specialists were cardiologists except in Russia, Saudi Arabia, and the UAE, where 15% of specialists were endocrinologists.

**Table 1:** Number of specialists and GPs who completed the survey

Country	Number of specialists (% of total)	Number of GPs (% of total)
Austria	20 (33%)	40 (67%)
Belgium	33 (55%)	27 (45%)
Colombia	20 (33%)	40 (67%)
Croatia	15 (50%)	15 (50%)
Czech Republic	20 (33%)	40 (67%)
Denmark	23 (38%)	37 (62%)
Portugal	15 (25%)	45 (75%)
Russia	33 (55%)	27 (45%)
Saudi Arabia	40 (67%)	20 (33%)
Switzerland	28 (47%)	32 (53%)
Turkey	30 (50%)	30 (50%)
United Arab Emirates	33 (55%)	27 (45%)

*GP* general/family physician

### Signs and Symptoms Associated with Statins

**Fig. 1:**
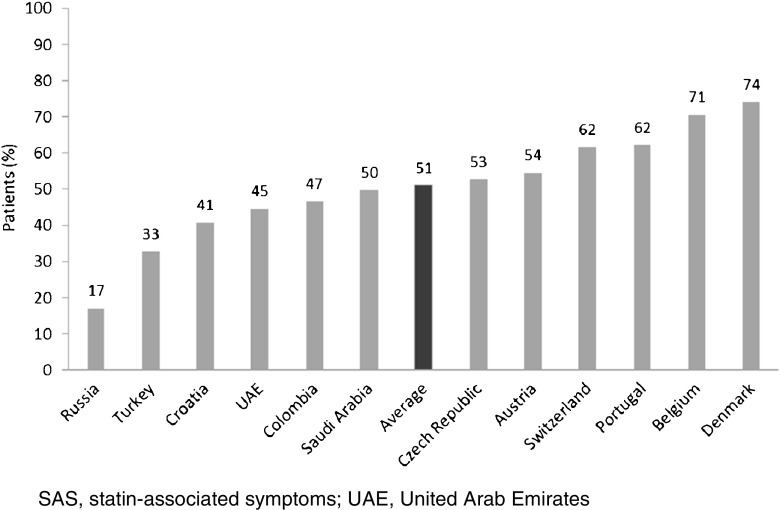
Patients (%) presenting with potential SAS who have muscle-related symptoms

Respondents across all countries except Russia reported that muscle-related symptoms were the most common symptom among patients presenting with potential SAS (range across all countries 17–74%; average of 51%) (Fig. 1). Clinicians in Russia reported that the most frequently seen sign or symptom was persistent elevation in transaminases (41% of patients) compared with 8–32% across the remaining countries.

Across the countries, an average of 15% of patients (range across all countries 9–24%) were reported to present with elevated levels of creatine kinase but no muscle symptoms; an average of 51% of clinicians routinely measured creatine kinase in all patients newly prescribed statins (range across all countries 35–67%). An average of 13% of patients (range across all countries 4–25%) were reported to present with other symptoms (e.g., gastrointestinal symptoms or alopecia). Patients were much less commonly reported to present with CNS effects (range across all countries 2–8%; average of 4% of patients).

### Minimum Criteria Used in Clinical Practice to Establish SAS in Patients with Muscle-Related Symptoms

Clinicians were asked to identify the minimum criteria that they use to establish SAS in patients presenting with muscle-related symptoms (i.e., SAMS). The following options suggested in the questionnaire were:

- discontinuation or lowering the statin dose to assess the effect on signs or symptoms, including when followed by rechallenge;
- modification of the statin regimen through change in the statin type, dose, or dosing schedule;
- evaluation of creatine kinase levels after discontinuation or lowering of the statin dose; and
- consideration of other causes for signs or symptoms.

### Rechallenge

**Fig. 2:**
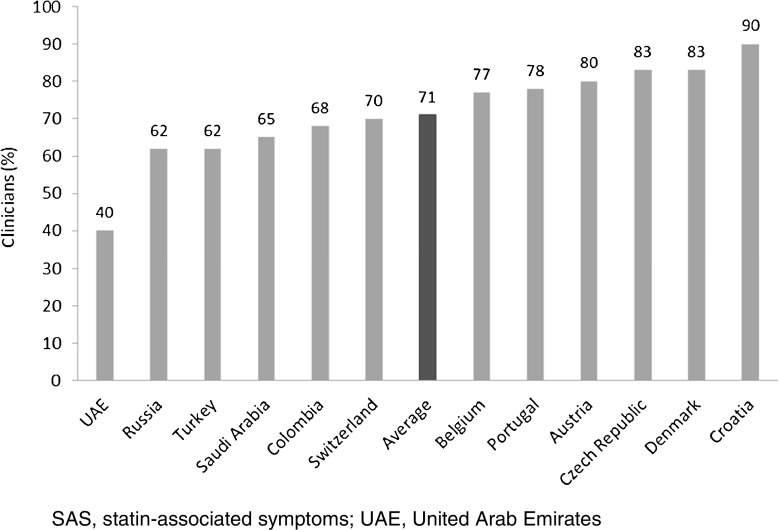
Clinicians (%) who rechallenge as a minimum requirement before considering muscle-related symptoms to be SAS

Rechallenge was defined as identifying whether a patient’s symptoms or signs improve or resolve when statin treatment is discontinued but recommence when a statin is restarted. It was reported as a minimum criterion by an average of 71% of clinicians (range across all countries: 40–90%) (Fig. 2). In most countries (9 of 12), specialists were more likely to require this criterion than GPs: on average 75% of specialists (range across all countries 42–95%) selected this criterion compared with 67% of GPs (range across all countries 37–95%). Those who did not report rechallenge with statins would usually either discontinue the statin or lower the dose to establish whether symptoms improve or resolve; only a small number of clinicians (range across all countries 0–15%) selected none of these options.

### Modification

**Fig. 3:**
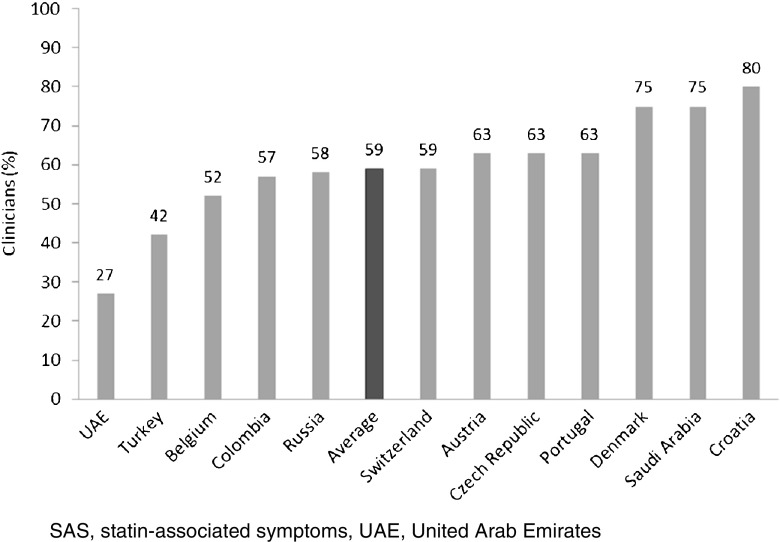
Clinicians (%) who try ≥2 alternative statins before considering muscle-related symptoms to be SAS

Modification of the statin regimen was commonly reported by clinicians as a minimum criteria used to establish SAS in patients presenting with muscle-related symptoms (i.e., SAMS). An average of 74% clinicians (range across all countries 32–90%) reported that they would try at least one alternative statin before considering a patient with muscle-related symptoms to have SAS; an average of 59% clinicians (range across all countries 27–80%) would try at least two alternative statins (Fig. 3). These criteria were selected by similar proportions of specialists and GPs: 72% of specialists would try at least one alternative statin and 59% would try at least two, compared with 76 and 58% of GPs. An average of 30% of clinicians (range across all countries 0–60%) would try at least one statin at a lower dose, at least one statin at an intermittent dose, and at least two alternative statins.

### Other Criteria

Two other proposed criteria were widely considered as a minimum requirement. An average of 86% of clinicians (range across all countries 77–100%) would recheck serum creatine kinase levels after stopping or modifying statin therapy in patients with previously elevated serum creatine kinase levels and potential SAS with muscle-related symptoms (i.e., SAMS).

Exclusion of other potential causes of symptoms is also part of recently proposed definitions of statin intolerance; [12, 16] this was selected as part of the minimum set of criteria for establishing SAS in patients with muscle-related symptoms by 57% of clinicians (range across all countries 15–87%). Similar proportions of specialists and GPs reported using each of these criteria.

### Minimum Criteria Used in Clinical Practice to Establish SAS in Patients with Persistent Elevation of Transaminases

In Russia, persistent elevation of transaminases was reported to be the most common presenting sign/symptom of potential SAS. For these patients, a higher proportion of clinicians required rechallenge, and would try at least two alternative statins, than for patients with muscle-related symptoms.

### Estimates of the Prevalence of SAS

Although this study was not designed as an epidemiological study, the prevalence of SAS was estimated based on clinicians’ responses: clinicians were asked what proportion of patients newly prescribed statins present with signs or symptoms that could be due to intolerance (i.e., SAS), and what proportion of these patients are later confirmed to be unable to tolerate statins at a dose below the label recommendation (according to the clinicians’ own criteria).

**Fig. 4:**
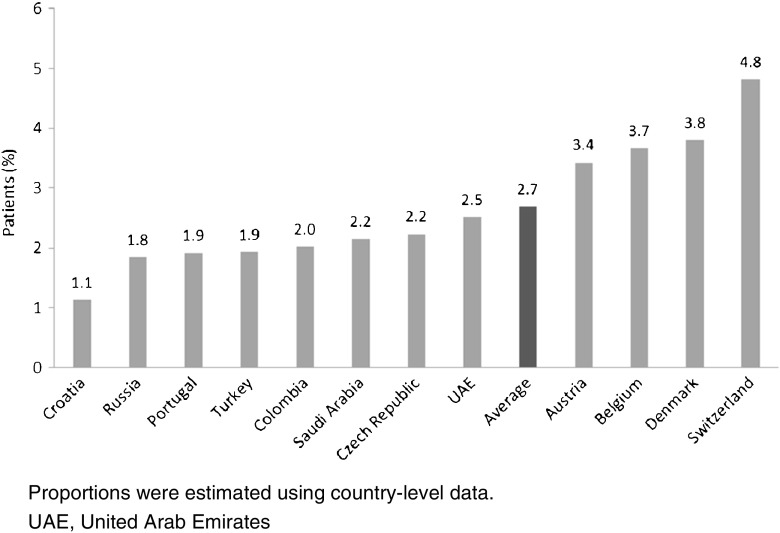
Estimated proportion of patients confirmed to have statin intolerance

An average of 12% of patients with hypercholesterolemia who are treated with statins across these 12 countries are reported to have signs and symptoms that may indicate SAS (range 7–21%); of these, an average of 23% (range across all countries 12–34%) are unable to tolerate statins at the recommended therapeutic dose. Therefore, it is estimated that an average of 2.7% of the total patient population (23% of the 12% of patients presenting with symptoms) are confirmed as being unable to tolerate statins at the recommended therapeutic dose (range across all countries 1.1–4.8%) (Fig. 4). This reported percentage was similar for specialists (0.5–5.3%, average 2.7%) and GPs (0.9–4.5%, average 3.3%).

Of the estimated 2.7% of patients confirmed to have SAS, an average of 55% was estimated to have muscle-related symptoms (i.e., SAMS). This ranged from 30% in Russia to 70% in Denmark, and was the most prevalent sign or symptom in all countries except Russia, where persistent elevation in transaminases was the most common symptom among patients confirmed as statin intolerant (47% of patients).

### Treatment Strategies for Patients with SAS

**Fig. 5:**
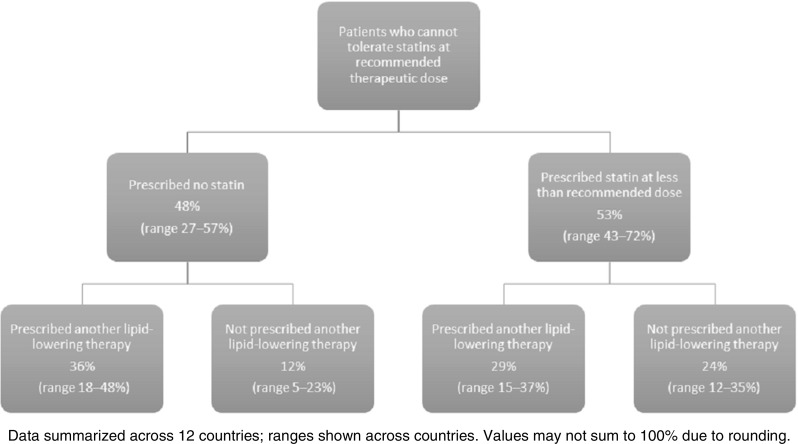
Treatment strategies for patients who cannot tolerate statins at the recommended therapeutic dose

Clinicians were asked how they treat patients at high cardiovascular risk who are unable to tolerate statins at the recommended therapeutic dose. Clinicians across all countries reported that an average of 53% of their high-risk patients with confirmed SAS continue to receive a lower-dose statin (range across all countries 43–72%; defined in the questionnaire as lower than the recommended daily or weekly therapeutic dose). An average of 65% of these patients would receive another non-statin lipid-lowering therapy with or without a concomitant statin (range across all countries 52–83%), and some patients (average 12%, range across all countries 5–23%) did not receive any statins or other non-statin lipid-lowering therapies. Figure 5 summarizes the treatment pathway inferred from clinicians’ responses across countries for patients who are unable to tolerate statins at the recommended therapeutic dose. There was little difference between specialists and GPs across countries in the use of lower-dose statins (on average 53 and 51% of their patients, respectively), their use of non-statin lipid-lowering therapy (30 and 29% of their patients, respectively), or the proportion of their patients who did not receive any statins or other non-statin lipid-lowering therapies (13 and 12%, respectively).

Clinicians were asked whether they used vitamin D supplementation to ameliorate SAS: 35% of respondents indicated that they did so, although use varied considerably between countries (13% in Croatia compared with 80% in the UAE).

### Use of Non-Statin Lipid-Lowering Therapies

Clinicians were asked which non-statin therapies they used most frequently for patients with confirmed SAS. Ezetimibe was the most common first-choice non-statin therapy used without a concomitant statin in 10 of 12 countries (first choice of 55% of clinicians across countries), but the choice of therapy differed between countries and between GPs and specialists (Table 2). Fibrates were the first choice of the majority of both specialists and GPs in Saudi Arabia and the UAE (listed therapies included fenofibrate, bezafibrate, ciprofibrate, and gemfibrozil); fibrates were the first choice of GPs in the Czech Republic and Turkey.

**Table 2:** First choices of non-statin monotherapy for patients with confirmed SAS

Country	Most common first-choice therapy (% of clinicians selecting as first choice)	
	Specialists	GPs
Austria	Ezetimibe (85%)	Ezetimibe (64%)
Belgium	Ezetimibe (85%)	Ezetimibe (63%)
Colombia	Ezetimibe (42%)	Ezetimibe (47%)
Croatia	Ezetimibe (62%)	Fibrates^a^ (43%)
Czech Republic	Ezetimibe (80%)	Fibrates^a^ (84%)
Denmark	Ezetimibe (91%)	Ezetimibe (91%)
Portugal	Ezetimibe (79%)	Ezetimibe (68%)
Russia	Ezetimibe (43%)	Ezetimibe (39%)
Saudi Arabia	Fibrates^a^ (66%)	Fibrates^a^ (53%)
Switzerland	Ezetimibe (89%)	Ezetimibe (81%)
Turkey	Ezetimibe (66%)	Fibrates^a^ (66%)
UAE	Fibrates^a^ (67%)	Fibrates^a^ (56%)

^a^Clinicians selected from a list of therapies including fenofibrate, bezafibrate, ciprofibrate, and gemfibrozil; there were differences in the frequency of selection of individual fibrates between countries

*GP* general/family physician, *UAE* United Arab Emirates

Clinicians in all countries reported similar choices of non-statin therapy for use in combination with lower-dose statin regimens (a daily or weekly dose lower than the recommended therapeutic dose).

### Alignment of Responses with Clinical Guidelines

The results of this survey align well with recent clinical practice guidelines for the management of patients presenting with potential SAMS (European Atherosclerosis Society Consensus Panel [10] and US National Lipid Association [11]). On average, the clinicians surveyed stated that they undertake the recommended measures to establish whether a patient’s symptoms are associated with statin use. The majority of clinicians stated that they tried statin washout followed by rechallenge, tried alternative statins, and would wish to exclude alternative causes of symptoms. Regarding the management of patients identified as statin intolerant, the clinicians stated that they use lower-dose statins where tolerated and also use non-statin lipid-lowering therapies (with ezetimibe as first choice) in order for patients to achieve their low-density lipoprotein cholesterol goal; both of these strategies are recommended in the guidelines.

For both identification and management of patients, however, there were differences between countries in whether the majority of clinicians’ practice aligned with these guidelines; this is discussed below.

## Discussion

This study extends our previous work [17] to an additional 12 countries. The aim was to elicit responses from clinicians who were experienced in managing patients with SAS, and to understand their routine clinical practice. As in the previous work, clinicians in most countries surveyed stated that they required relatively stringent criteria to be fulfilled to establish SAS (typically SAMS or other signs/symptoms of intolerance), including rechallenging after discontinuation to identify the association of symptoms, use of alternative statins, and exclusion of other potential causes of signs/symptoms.

The estimated prevalence of confirmed SAS was less than 3% in 8 of the 12 countries, which is lower than the prevalence reported in observational studies of about 10% [5–8]. The estimate from our study is based on clinicians’ own definitions, and may include patients who continue to receive a lower dose of statin if the clinician considers them to have SAS. This suggests that clinicians are usually stringent in excluding other possible causes when evaluating patients with signs or symptoms of potential SAS. Variation between countries may reflect the actual prevalence of signs and symptoms (potentially influenced by the dosage and type of statin prescribed), and the attitude of patients towards side-effect reporting.

Clinicians’ responses suggest that approximately half of high-risk patients continue to receive a lower-dose statin (with unknown adherence), and the majority are also prescribed an alternative lipid-lowering therapy: a pattern consistent with the previous countries surveyed. However, a proportion of patients (estimated to be over 10% in 7 of the 12 countries surveyed) do not receive any treatment.

The objective of this work was not to make comparisons among countries, but to identify practice patterns on a country level. However, some differences between countries in both the identification and management of patients with SAS are clear:

- As in the previous survey, muscle symptoms were most commonly reported as a presenting sign or symptom of statin side effects, particularly in Western European countries. Elsewhere, other signs/symptoms were considered more frequent; persistent elevation of transaminases was reported to be the most common presenting sign or symptom in Russia. This may reflect the frequency of monitoring: in Russia, over 90% of clinicians reported testing for transaminase elevation in all patients newly prescribed statins, compared with an average of 79% across all countries (range 60–92%). Other considerations may be clinical (statin dosing, genetic factors, comorbidities such as viral hepatitis and alcohol abuse) or cultural factors.
- The stringency of the criteria that had to be met for a patient to be considered to have SAS (typically SAMS or other signs/symptoms of intolerance) was similar across most countries; however, notably fewer clinicians in the Russia, Turkey, and the UAE reported requiring rechallenge and fewer reported trying multiple statins.
- Unlike in the previous survey, where ezetimibe was the most commonly selected therapy for use in patients with SAS in all countries, there were some regional differences in the choice of therapy; for example, fibrates were the first-choice alternative to statins for clinicians in the two Middle Eastern countries. This choice of therapy may be influenced by the local prevalence of mixed dyslipidemia, country- and region-specific clinical evidence, or local reimbursement, marketing, and experience of particular agents. Differences between specialists and GPs may reflect familiarity or local reimbursement restrictions that require specialist prescribing.

### Limitations

The anonymous nature of the data collection and analyses aims to ensure that the results of this survey can provide insight into clinical practice for patients with SAS in each of the countries surveyed. However, the restriction of participants to those who managed a certain number of patients with hypercholesterolemia and with statin intolerance is a limitation of this work, and may mean that the responses cannot be generalized to all clinicians in their country. There is also a bias towards clinicians willing to participate in such research. The survey also relies on clinicians’ responses and hence their recall, rather than using patient-level data to identify the decisions they actually make in practice. Comparison with the results of an alternative methodology (e.g., review of medical records), would be informative to understand the extent of correlation between clinician-reported practice and patient-level records. We recognize that patient and physician perspectives concerning statin intolerance vary. The use of a standardized validated tool has been proposed to improve the accuracy of identification of statin versus non-statin-associated muscle symptoms [18].

## Conclusions

In line with the findings of the previous work, this survey provides evidence that clinicians have a somewhat uniform way of defining SAS. In general, stringent criteria are used to establish whether the relationship between statins and signs or symptoms is causal and most clinicians strive to continue statin treatment for their patients at high cardiovascular risk where possible. The expanded geographic scope provides a broader understanding of similarities and differences across countries: differences between countries were identified in the most frequently presented sign or symptom of SAS, and in the choice of non-statin lipid-lowering therapies.

Recently there has been increased interest in the management of patients with SAS, exemplified by numerous guidelines at a national and international level, [9–12, 15, 16] and the recent publication of a clinical index for identification of SAMS [18]. This, coupled with increasing treatment options and evidence of the effectiveness of alternative lipid-lowering therapies, suggests that control of hypercholesterolemia in such patients can be improved. Only a small minority of all patients prescribed statins are not able to tolerate any statin dose; however, given the established beneficial effect of statins [4] and potential harm of down-titrating or discontinuing statin therapy [14], it is essential that clinicians do utilize stringent criteria, such as rechallenge, and try treatment strategies such as lower- or intermittent-dosing, in order to ensure statin use in the largest possible proportion of patients at high cardiovascular risk.

## Electronic supplementary material

Supplementary material 1 (DOCX 22 kb)
