# Supplementary material for: Identification and Management of Statin-Associated Symptoms in Clinical Practice: Extension of a Clinician Survey to 12 Further Countries
Source: Cardiovasc Drugs Ther. 2017 May 3;31(2):187–95. doi: 10.1007/s10557-017-6727-0 (PMC5427112; doi:10.1007/s10557-017-6727-0)
Supplement: Supplementary file 1 — 1 (DOCX 22 kb) [file 10557_2017_6727_MOESM1_ESM.docx]

Summary of the questionnaire

| Section | Purpose |
| --- | --- |
| Screener | - To identify clinicians with the relevant knowledge and experience to complete the survey - To collect information on specialty type and clinical practice to assess the representativeness of the survey sample |
| Identification of patients with statin-associated symptoms | - To identify various statin-associated symptoms - To understand the minimum criteria used to identify patients with statin-associated symptoms at high risk of a CVD event - To estimate the proportion of patients with statin-associated symptoms in the clinician’s caseload, based on the criteria identified |
| Treatment of patients with statin-associated symptoms | - To identify factors that influence treatment decisions - To establish the aim and duration of treatment - To identify the standard of care - To establish whether patients are re-challenged with statins to confirm statin-associated symptoms |
| Case study 1 | - To explore the treatment steps for a typical high-risk patient between presenting with muscle-related symptoms and switching to a non-statin lipid-lowering therapy - To identify clinicians’ first choice of non-statin lipid-lowering therapy |
| Case study 2 | - To determine how LDL-C levels influence clinicians’ decisions to add further therapies to a low-dose statin - To establish what further steps would be taken to optimize treatment for a patient receiving a low-dose statin regimen |

Abbreviations: CVD, cardiovascular disease; LDL-C, low-density lipoprotein cholesterol
